# Evaluation of contezolid’s cerebrospinal fluid concentration and safety in tuberculous meningitis patients

**DOI:** 10.1128/spectrum.02399-25

**Published:** 2026-03-13

**Authors:** Mailing Huang, Yujin Wang, Tao Chen, Yu Lu, Naihui Chu, Wenjuan Nie

**Affiliations:** 1 Department of Tuberculosis, Beijing Chest Hospital, Capital Medical University, Beijing, China; 2 Beijing Tuberculosis and Thoracic Tumor Institute, Beijing, China; 3 Shanghai Institute of Infectious Disease and Biosecurity, Fudan University, Shanghai, China; 4 Drug Research Laboratory, Beijing Chest Hospital, Capital Medical University, Beijing, China; The University of Texas at Tyler, Tyler, Texas, USA

**Keywords:** contezolid, cerebrospinal fluid concentration, tuberculous meningitis

## Abstract

Tuberculous meningitis (TBM) is the most severe form of tuberculosis (TB) with high mortality. This study evaluated the cerebrospinal fluid (CSF) concentration and safety of the novel oxazolidinone contezolid compared to linezolid in TBM patients. In this randomized prospective study, 10 TBM patients received either a linezolid-containing (*n* = 5) or contezolid-containing (*n* = 5) anti-TB regimen. CSF and blood concentrations were measured at 2 and 6 h post-dose. Peak (2 h) and trough (6 h) concentrations, area under the concentration-time curve (AUC), and safety were assessed. Contezolid CSF concentrations exceeded the *Mycobacterium tuberculosis* (Mtb) MIC (0.5 μg/mL) at 2 h (median: 1.0806 μg/mL, range: 0.9295–1.3165 μg/mL) but declined by 6 h (median: 0.7920 μg/mL, range: 0.1867–1.0194 μg/mL). Linezolid CSF concentrations were significantly higher than contezolid at both 2 h (median: 3.251 μg/mL, range: 1.9545–4.9636 μg/mL; *P* = 0.008) and 6 h (median: 1.623 μg/mL, range: 0.941–1.765 μg/mL; *P* = 0.016), remaining above MIC in all samples at 6 h. The mean CSF AUC for contezolid (4.637 μg·h/mL, 95% CI: 3.599–5.675) was significantly lower than that for linezolid (12.537 μg·h/mL, 95% CI: 7.8797–17.277; *P* = 0.008). Blood concentrations were higher than CSF for both drugs at all time points. No serious drug-related adverse events occurred. Contezolid effectively penetrates the blood-CSF barrier in TBM patients, achieving CSF concentrations above the MIC for Mtb. Although its CSF exposure was significantly lower than linezolid, its demonstrated penetration and safety profile suggest contezolid warrants further investigation as a potential treatment option for drug-resistant TBM.

**IMPORTANCE:**

Tuberculous meningitis (TBM) is the deadliest form of tuberculosis, especially difficult-to-treat drug-resistant TBM. Finding new, effective, and safe medicines is critical. This study provides evidence in TBM patients that a newer antibiotic, contezolid, successfully reaches the infection site in cerebrospinal fluid (CSF) at levels expected to kill *Mycobacterium tuberculosis*. While linezolid achieved higher levels in CSF, contezolid still reached concentrations predicted to be effective and caused no serious side effects in our study. This is important because contezolid might offer a safer alternative to linezolid, which can have significant long-term toxicity. These promising results suggest contezolid could become a valuable new weapon against refractory drug-resistant TBM, potentially saving lives where current options are limited or too toxic.

## INTRODUCTION

Tuberculosis (TB) remains the leading cause of death from infectious diseases globally (1). Tuberculous meningitis (TBM), a central nervous system manifestation of TB, is characterized by subacute or chronic inflammation of the meninges, brain parenchyma, and cerebral vasculature caused by *Mycobacterium tuberculosis* (Mtb) infection (2). Treatment of drug-resistant TBM is particularly challenging due to the limited availability of oral second-line medications capable of penetrating the blood-cerebrospinal fluid (CSF) barrier.

Linezolid is widely used due to its excellent blood-CSF barrier penetration but is associated with significant adverse effects, including myelosuppression and optic neuropathy (3). Contezolid, a next-generation oxazolidinone, has demonstrated comparable anti-TB activity with a potentially improved safety profile compared to linezolid (4). Recently, our team has released the “Chinese Expert Consensus on Contezolid Anti-tuberculosis” in China, further emphasizing the clinical value of the drug. However, there is still a significant gap in research on this special population of TBM (3). Based on this, the aim of this study is to systematically evaluate the CSF concentration distribution of contezolid in TBM patients and its pharmacokinetic differences with linezolid, clarify whether it can achieve and maintain effective levels above the minimum inhibitory concentration (MIC) through blood-CSF barrier, and evaluate its safety characteristics, providing key evidence for optimizing drug-resistant TBM treatment plans.

## MATERIALS AND METHODS

### Subject selection

The work was performed in Beijing Chest Hospital. This study included hospitalized patients with a definite or probable TBM using the uniform clinical case definition described by Marais and colleagues (5) within the past 3 months: definite TBM is a clinical syndrome or sign suggestive of meningitis, with bacteriological evidence by any of the smear microscopy, culture, or Xpert from CSF; probable TBM requires a diagnostic score of 12 or above with cerebral imaging available, whereas a diagnostic score of 10 or above was required with cerebral imaging unavailable. Inclusion criteria included patients with a definite or probable TBM who were willing to participate with signed informed consent and effective contraception for both sexes during and 1 month after the study. Exclusion criteria included corticosteroid or immunosuppressant use within 90 days before screening; pregnancy, puerperium, or breastfeeding; allergy to or known hypersensitivity, severe adverse reactions to contezolid or linezolid; drug resistance to contezolid or linezolid; unsuitability for study participation as assessed by the investigator; and potential health risks from participation or inability to comply with study visits and assessments, as determined by the investigator.

### Study design

This randomized prospective study compared the pharmacokinetic characteristics of contezolid and linezolid in TBM patients, focusing on CSF drug concentrations, including peak level and area under the concentration-time curve (AUC), and assessed the safety of both drugs. The lottery method was used for randomization, and no blind method was used.

Statistical analysis was performed using R version 4.3.3 and SPSS version 22.0. Fisher’s exact test was used for the comparison of categorical data, and the Mann-Whitney *U*-test was used for the comparison of continuous data. Differences with a *P* value of 0.05 or less were considered statistically significant.

### Drug administration

Patients were randomly assigned to the contezolid or linezolid group. The contezolid group received 800 mg orally twice daily 30 min after breakfast (8 a.m.) and supper (8 p.m.), and the linezolid group received 600 mg orally once daily 30 min after breakfast (8 a.m.). Concurrently, all the patients received anti-TB treatment with regimens containing isoniazid (300 mg), rifampicin (450 mg, body weight <50 kg; 600 mg, body weight 50 kg), pyrazinamide (1,500 mg), and ethambutol (750 mg) once daily, as recommended by the WHO, with direct observation by research staff and patient diaries with specific instructions. Strict adherence to the drug regimen was required, with blood and CSF samples collected at specified time points.

### Sample collection and analysis

Seven days after antituberculosis treatment, blood (2 mL from the median cubital vein) and cerebrospinal fluid (2 mL via lumbar puncture) samples were collected 2 and 6 h post-dosage. Samples were immediately placed in pre-chilled centrifuge tubes, centrifuged at 3,000 rpm for 10 min at 4°C to separate plasma and the CSF supernatant, transferred to new tubes, and stored at −80°C. Drug concentrations were measured by high-performance liquid chromatography–tandem mass spectrometry using a validated method to ensure accuracy and reliability.

## RESULTS

### Characteristics of the participants

Of the 10 cases enrolled in this study, 5 cases were diagnosed as definite TBM, while the other 5 cases were diagnosed as probable TBM, according to the uniform clinical case definition described by Marais and colleagues (Table 1) (5). Among them, 5 patients (including 3 with definite TBM and 2 with probable TBM) received a linezolid-containing antituberculosis regimen, while the other 5 (including 2 with definite TBM and 3 with probable TBM) received a contezolid-containing regimen. The median age of all the 10 patients was 47.74 years (range: 29–65 years). Similar to other tuberculosis patients, all the TBM patients included in the study had a lower body mass index (BMI). All the patients were not infected with HIV. As the anti-TB treatment began, all the patients received treatment with glucocorticoids (dexamethasone 0.3–0.4 mg/kg). The leukocyte count in the CSF of all patients was elevated. Before treatment, one patient in the linezolid group exhibited thrombocytosis (451 × 10⁹/L), which normalized after anti-TB therapy. Additionally, one patient in the linezolid group and two patients in the contezolid group had anemia (100, 116, and 78 g/L, respectively). After anti-TB treatment, hemoglobin levels in all three patients returned to normal. There were no statistically significant differences in terms of diagnostic classification, sex, age, BMI, HIV status, prior TB treatment, corticosteroid use, leukocyte count in CSF, neutrophilic granulocyte percentage in CSF, leukocyte count in blood, platelet count, hemoglobin level, aspartate aminotransferase, total bilirubin, urea nitrogen, creatinine, and ECG QTc interval between the linezolid and contezolid groups. Although the alanine aminotransferase level in the contezolid group was a little lower, all values were within the normal reference range (Table 1).

**TABLE 1:** The demographic and clinical characteristics of the participants^*a*^

Characteristics	All participants (*N* = 10)	Linezolid group (*n* = 5)	Contezolid group (*n* = 5)	*P* value^*b*^
Definite TBM	5/10 (50.00%)	3/5 (60.00%)	2/5 (40.00%)	1.000
Male	5/10 (50.00%)	2/5 (40.00%)	3/5 (60.00%)	1.000
Age (year)	47.74 (38.93–56.54)	42.54 (36.54–48.54)	58.03 (44.99–71.07)	0.602
BMI (kg/m^2^)	17.86 (17.41–18.45)	17.85 (17.44–18.82)	17.86 (16.99–18.31)	0.690
HIV	0/10 (0.00%)	0/5 (0.00%)	0/5 (0.00%)	1.000
Prior TB treatment	5/10 (50.00%)	2/5 (40.00%)	3/5 (50.00%)	1.000
Corticosteroid use	10/10 (100.00%)	5/5 (100.00%)	5/5 (100.00%)	1.000
Leukocyte in CSF (/μL)	432.50 (286.50–508.25)	439.00 (258.00–530.50)	429.00 (242.00–522.00)	0.841
N% in CSF	7.00 (5.75–9.25)	6.00 (5.50–9.50)	8.00 (5.50–10.00)	0.841
Leukocyte in blood (×10^9^/L)	7.74 (6.56–8.92)	7.52 (6.88–8.16)	8.24 (6.43–10.05)	0.754
Platelet (×10^9^/L)	319.69 (270.55–368.83)	324.36 (314.48–334.24)	234.44 (184.24–284.64)	0.347
Hemoglobin (g/L)	134.36 (123.85–144.88)	134.92 (128.53–141.3)	123.6 (113.1–134.1)	0.347
ALT (U/L)	10.71 (3.49–17.93)	17.13 (8.73–25.52)	4.00 (3.21–4.79)	0.045
AST (U/L)	23.37 (17.66–29.07)	24.00 (16.34–31.66)	22.74 (17.99–27.48)	0.917
Total bilirubin (μmol/L)	7.66 (6.2–9.11)	7.36 (4.32–10.4)	7.95 (6.57–9.33)	0.754
Urea nitrogen (mmol/L)	4.06 (3.34–4.77)	3.77 (3.3–4.24)	4.14 (3.26–5.02)	0.175
Creatinine (μmol/L)	59.81 (52.89–65.44)	61.19 (52.31–66.22)	58.44 (54.64–63.11)	0.835
ECG QTc interval (ms)	421.50 (394.85–433.51)	413.72 (402.27–429.81)	429.29 (391.24–434.75)	0.835

^*a*^ Data are presented as medians (Q1–Q3) or *n*/*N* (%), unless otherwise stated. ALT, alanine aminotransferase; AST, aspartate aminotransferase; BMI, body mass index; N%, neutrophilic granulocyte percentage.

^*b*^ Comparison between the linezolid group and the contezolid group. Fisher’s exact test was used for diagnosis, gender, HIV, prior TB treatment and corticosteroid use comparison between Linezolid and Contezolid group, and the Mann-Whitney U-test was used for the comparison of age, BMI, leukocyte count in CSF, neutrophilic granulocyte percentage in CSF, leukocyte in blood, platelet, hemoglobin, ALT, AST, total bilirubin, urea nitrogen, creatinine, and ECG QTc interval between the linezolid and contezolid groups.

### Drug distribution in blood and CSF

After oral administration of contezolid, the CSF concentration at 2 h ranged from 0.9295 to 1.3165 μg/mL (median [Q1–Q3]: 1.0806 [1.0643–1.1567] μg/mL), significantly exceeding the MIC for *Mycobacterium tuberculosis* (0.5 μg/mL), with all samples meeting the target (100%). By 6 h, the concentration declined to 0.1867–1.0194 μg/mL (0.792 [0.4567–0.8166] μg/mL) but remained detectable. Post-dose blood concentrations of contezolid were 8.298–25.784 μg/mL at 2 h (12.594 [9.241–14.650] μg/mL), dropping to 3.858–11.21 μg/mL at 6 h (5.59 [5.394–6.056] μg/mL). For linezolid, the CSF concentration at 2 h was 1.9545–4.9636 μg/mL (3.251 [2.4154–3.442] μg/mL), surpassing its MIC (0.5 μg/mL), with a 100% target attainment. At 6 h, the concentration decreased to 0.941–1.765 μg/mL (1.623 [1.222–1.752] μg/mL), still meeting the target in all samples (100%). Blood concentrations of linezolid were 7.8651–24.666 μg/mL at 2 h (21.7999 [13.1341–22.9139] μg/mL) and declined to 5.8773–10.3957 μg/mL at 6 h (7.9311 [7.3385–9.7314] μg/mL).

**Fig 1:**
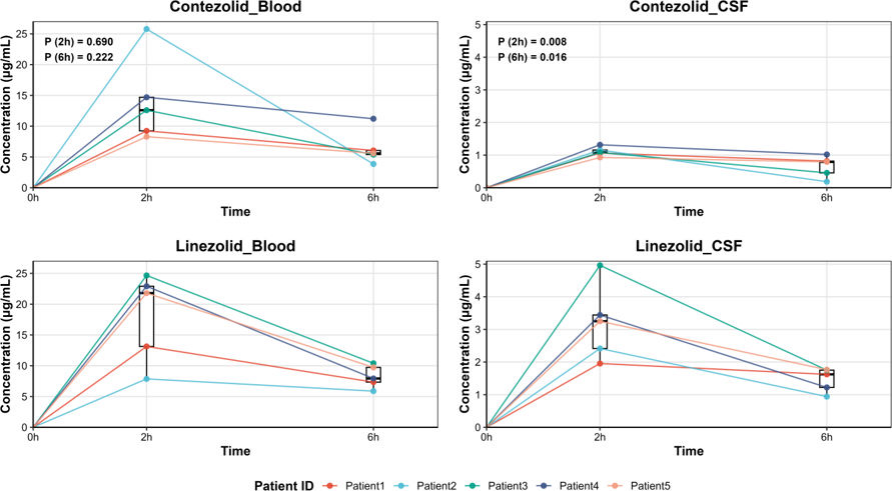
Blood and CSF drug concentrations of contezolid and linezolid at 2 and 6 h. Five different colors represent five patients. CSF, cerebrospinal fluid. Blood concentrations of contezolid and linezolid were significantly higher at 2 h than at 6 h after administration. However, the blood concentrations of both drugs at 2 and 6 h were similar. In contrast, the concentration of contezolid in CSF was significantly lower than that of linezolid at 2 or 6 h after administration. Blood concentrations were higher than CSF for both drugs at all time points.

Mann-Whitney *U*-test on CSF concentrations showed significant differences between contezolid and linezolid at both 2 h (*P* = 0.008) and 6 h (*P* = 0.016), while there was no significant difference in blood concentrations between contezolid and linezolid at both 2 h (*P* = 0.690) and 6 h (*P* = 0.222) (Fig. 1).

**Fig 2:**
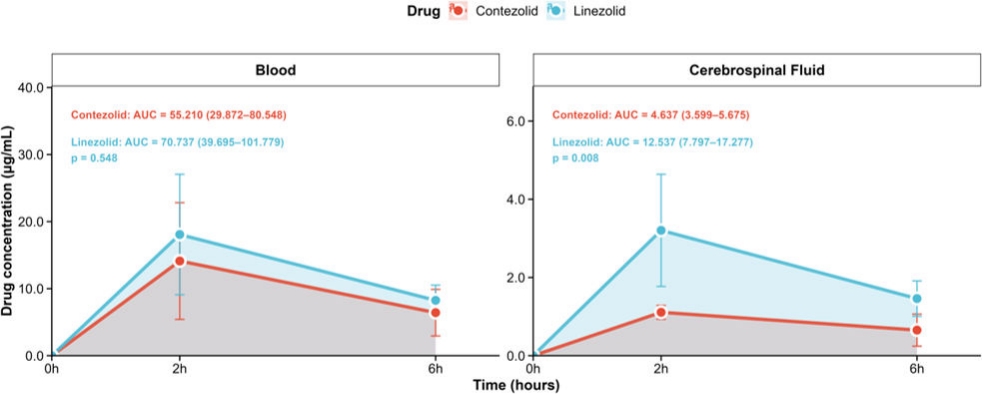
Area under the concentration-time curve (pharmacokinetic AUC) plots of contezolid and linezolid on blood and cerebrospinal fluid. The red line represents the drug concentration for contezolid, while blue represents the drug concentration for linezolid. Data are presented as means (95% CI). Mann-Whitney *U*-test was used for the comparison of CSF or blood AUC between contezolid and linezolid. The mean blood AUC for contezolid was similar to that for linezolid, while the mean CSF AUC for contezolid was lower than that for linezolid.

The mean CSF AUC for contezolid was 4.637 (95% CI: 3.599–5.675) μg·h/mL, and that for linezolid was 12.537 (95% CI: 7.8797–17.277) μg·h/mL. The difference was statistically significant (*P* = 0.008), indicating significantly higher CSF exposure with linezolid. While the mean blood AUC for contezolid was 55.210 (95% CI: 29.872–80.548) μg·h/mL and that for linezolid was 70.737 (95% CI: 39.695–101.779) μg·h/mL, there was no difference between contezolid and linezolid (*P* = 0.548) (Fig. 2).

### Safety evaluation

**Fig 3:**
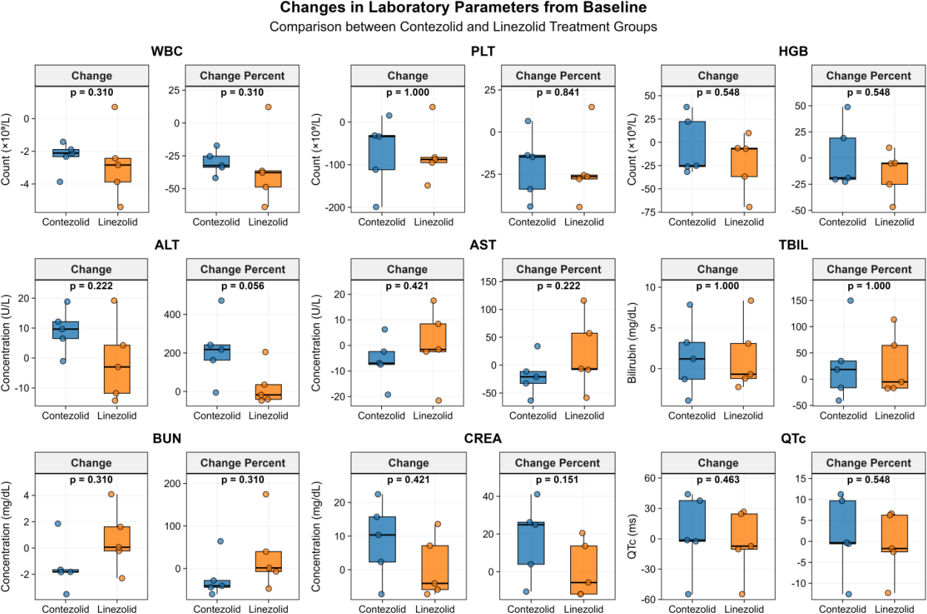
Changes in laboratory parameters from baseline comparison between contezolid and linezolid treatment groups. ALT, alanine aminotransferase; AST, aspartate aminotransferase; BIL, total bilirubin; BUN, blood urea nitrogen; CREA, creatinine; HGB, hemoglobin; PLT, platelet; WBC, leukocyte. Change means the value of post-treatment minus pre-therapy. Change percent means the change divided by the value of pre-therapy. Mann-Whitney *U*-test was used for the comparison of WBC, PLT, HGB, ALT, AST, TBIL, BUN, CREA, and ECG QTc interval between the linezolid and contezolid groups. There was no significant difference in the changes in WBC, PLT, HGB, ALT, AST, TBIL, BUN, CREA, and ECG QTc interval before and after treatment between patients treated with linezolid and those treated with contezolid.

Two weeks after taking linezolid/contezolid, we conducted a safety evaluation. No patients developed optic neuropathy or peripheral neuropathy. There were no statistically significant differences in leukocyte, platelet, hemoglobin, alanine aminotransferase, aspartate aminotransferase, total bilirubin, urea nitrogen, creatinine, and ECG QTc interval between the two groups before and after treatment (*P* > 0.05) (Table S1). Similarly, there were no statistically significant differences in the changes in these indicators before and after treatment in both groups (*P* > 0.05) (Table S1; Fig. 3). No serious drug-related adverse events were reported during the study. Neither of the two groups of patients experienced bone marrow suppression, liver or kidney function damage, or QTc prolongation.

## DISCUSSION

TBM, the most severe TB form, constitutes 1%–5% of all TB cases and 5%–10% of extrapulmonary TB cases (4). It has a poor prognosis, with untreated cases having a nearly 100% mortality rate and 30%–50% of survivors developing severe neurological sequelae, even after antituberculosis treatment (5–7). A major challenge in treating drug-resistant TBM is the limited availability of drugs that can effectively penetrate the blood-CSF barrier.

Linezolid, a key drug for rifampicin-resistant TB, is crucial in TBM treatment due to its good blood-CSF barrier penetration. Studies indicate that including linezolid in treatment regimens significantly improves outcomes for severe and rifampicin-resistant TBM cases (8, 9). However, its adverse effects, such as myelosuppression, optic nerve damage, and peripheral neuritis, often lead to treatment discontinuation, posing a challenge for TBM treatment, which requires a prolonged therapy.

Contezolid, a novel oxazolidinone, shows anti-TB activity comparable to linezolid and has the potential to be an alternative treatment for rifampicin-resistant TB (10). Pharmacokinetic studies indicate that contezolid reaches peak blood concentrations approximately 2.5 h post-administration and has a protein binding rate of 90%. Although data on its blood-CSF barrier penetration, particularly on central nervous system TB, are limited, case reports have demonstrated its clinical potential. Guo et al. reported a case of a 32-year-old woman with TBM who was switched to contezolid (800 mg every 12 h) due to adverse effects from a multi-drug anti-TB regimen that included linezolid (11). At weeks 7 and 11 of treatment, the serum and CSF contezolid concentrations were 9.64 and 0.54 mg/L and 9.36 and 1.15 mg/L, respectively, with corresponding CSF-to-serum concentration ratios of 0.056 and 0.123. This suggests that CSF contezolid concentrations exceeded the MIC for Mtb and approached the unbound drug concentration in serum. Similarly, Xu et al. reported a 69-year-old woman who was switched from linezolid (600 mg once daily) to contezolid (400 mg every 12 h) due to intolerance (12). After nearly 8 months of treatment, the patient’s meningitis symptoms resolved; her test results improved; and linezolid-related adverse effects were alleviated.

The pharmacokinetic analysis in this study shows that at 2 and 6 h post-administration, CSF contezolid concentrations were about 10% of the corresponding blood levels. Given its plasma protein binding rate of approximately 90%, this indicates that nearly all unbound contezolid in the blood penetrates the blood-CSF barrier, highlighting its strong penetration ability. The CSF contezolid concentrations at 2 h post-administration in all patients exceeded the MIC of 0.5 μg/mL for the clinical isolate, and in over half of the patients, the CSF contezolid concentrations at 6 h post-administration still exceeded the MIC of 0.5 μg/mL. This indicates that there are sufficient intracranial drug levels to inhibit Mtb activity.

All participants were treated with linezolid or contezolid, simultaneously containing isoniazid, rifampicin, pyrazinamide, and ethambutol in our study. Approximately 95% of contezolid is catalyzed by flavin monooxygenase 5 (FMO5) and the reductase in the cytoplasm of liver; the main metabolites have no antibacterial activity. Less than 5% of the original drug remaining is excreted through urine and feces. No other drugs that can metabolize or inhibit/induce the activity of FMO5 have been observed; therefore, there was no drug interaction with contezolid in our study.

This study analyzed contezolid concentrations in both blood and CSF in five patients. However, there were some limitations: the small sample size, missing pre-dose sample Cmin, and lack of long observation for the efficacy and safety evaluation. Future studies with larger sample sizes, more time points for CSF drug concentration, and systematic long-term follow-up to fully assess the pharmacokinetic profile and confirm the efficacy and safety of contezolid in treating drug-resistant TBM are needed.

## Data Availability

The original contributions presented in the study are included in Table S1. Further inquiries can be directed to the corresponding author.

## SUPPLEMENTAL MATERIAL

Table S1 spectrum.02399-25-s0001.docx Comparison of laboratory parameter changes between contezolid and linezolid treatment groups.

ASM does not own the copyrights to Supplemental Material that may be linked to, or accessed through, an article. The authors have granted ASM a non-exclusive, world-wide license to publish the Supplemental Material files. Please contact the corresponding author directly for reuse.
