# Supplementary material for: Evaluation of contezolid’s cerebrospinal fluid concentration and safety in tuberculous meningitis patients
Source: Microbiol Spectr. 2026 Mar 13;14(4):e02399-25. doi: 10.1128/spectrum.02399-25 (PMC13055287; doi:10.1128/spectrum.02399-25)
Supplement: Table S1 — Comparison of laboratory parameter changes between contezolid and linezolid treatment groups. [file spectrum.02399-25-s0001.docx]

Supplemental table S1. Comparison of Laboratory Parameter Changes Between Contezolid and Linezolid Treatment Groups

| Variables | | ALL | Linezolid group | Contezolid group | p value |
| --- | --- | --- | --- | --- | --- |
| WBC | Pretherapy | 7.74 [6.05-8.41] | 7.52 [6.88-8.16] | 8.24 [6.43-10.05] | 0.754 |
|  | Posttreatment | 4.46 [3.80-6.75] | 4.24 [4.08-4.68] | 6.82 [3.71-6.89] | 0.531 |
|  | Change | -2.38 [-3.61--1.94] | -2.84 [-3.88--2.43] | -2.11 [-2.33--1.89] | 0.310 |
|  | Change Percent（%） | -35.09 [-40.84--27.06] | -37.77 [-48.74--36.43] | -32.44 [-33.75--25.27] | 0.310 |
| PLT | Pretherapy | 319.69 [270.55-368.83] | 324.36 [314.48-334.24] | 234.44 [184.24-284.64] | 0.347 |
|  | Posttreatment | 234.38 [202.57-251.28] | 241.50 [227.27-265.08] | 217.95 [197.44-249.71] | 0.531 |
|  | Change | -85.31 [-107.70--32.11] | -87.75 [-95.29--82.86] | -33.83 [-111.83--31.53] | 1.000 |
|  | Change Percent（%） | -25.99 [-32.40--14.02] | -26.44 [-27.86--25.55] | -14.75 [-33.91--13.77] | 0.841 |
| HGB | Pretherapy | 134.36 [123.85-144.88] | 134.92 [128.53-141.3] | 123.6 [113.1-134.1] | 0.347 |
|  | Posttreatment | 110.34 [108.27-124.48] | 109.71 [109.36-127.53] | 110.97 [107.91-115.34] | 1.000 |
|  | Change | -16.10 [-30.30-5.82] | -6.96 [-36.87--6.28] | -25.24 [-26.09-22.21] | 0.548 |
|  | Change Percent（%） | -12.10 [-22.13-6.26] | -5.16 [-25.15--4.69] | -19.04 [-20.42-19.14] | 0.548 |
| ALT | Pretherapy | 10.71 [3.49-17.93] | 17.13 [8.73-25.52] | 4 .00[3.21-4.79] | 0.045 |
|  | Posttreatment | 16.70 [14.60-18.20] | 16.34 [15.98-17.05] | 17.73 [13.67-18.36] | 1.000 |
|  | Change | 5.40 [-2.51-11.53] | -2.99 [-11.79-4.29] | 9.67 [6.51-12.15] | 0.222 |
|  | Change Percent（%） | 99.18 [-14.44-214.51] | -17.45 [-40.88-35.60] | 217.74 [162.75-241.75] | 0.056 |
| AST | Pretherapy | 23.37 [17.66-29.07] | 24.00 [16.34-31.66] | 22.74 [17.99-27.48] | 0.917 |
|  | Posttreatment | 22.79 [16.05-25.77] | 23.17 [22.40-27.99] | 18.37 [15.28-24.66] | 0.403 |
|  | Change | -2.42 [-7.33-4.29] | -1.60 [-2.46-8.45] | -6.96 [-7.46--2.39] | 0.421 |
|  | Change Percent（%） | -9.80 [-29.86-23.85] | -6.67 [-8.08-57.40] | -21.03 [-32.81--11.51] | 0.222 |
| TBIL | Pretherapy | 7.66 [6.2-9.11] | 7.36 [4.32-10.4] | 7.95 [6.57-9.33] | 0.754 |
|  | Posttreatment | 9.41 [6.92-12.46] | 10.94 [7.89-12.31] | 7.73 [6.65-12.51] | 0.753 |
|  | Change | 0.26 [-1.28-3.19] | -0.68 [-1.21-3.09] | 1.20 [-1.30-3.22] | 1.000 |
|  | Change Percent（%） | 6.57 [-16.88-56.95] | -5.23 [-17.06-64.38] | 18.38 [-16.35-34.66] | 1.000 |
| BUN | Pretherapy | 4.06 [3.34-4.77] | 3.77 [3.3-4.24] | 4.14 [3.26-5.02] | 0.175 |
|  | Posttreatment | 3.35 [2.46-4.72] | 3.83 [2.88-5.65] | 2.42 [2.32-4.70] | 0.209 |
|  | Change | -0.93 [-1.83-1.22] | 0.06 [-0.22-1.61] | -1.82 [-1.83--1.65] | 0.310 |
|  | Change Percent（%） | -17.56 [-43.11-30.29] | 1.59 [-7.10-39.85] | -40.54 [-43.96--28.02] | 0.310 |
| CREA | Pretherapy | 59.81 [52.89-65.44] | 61.19 [52.31-66.22] | 58.44 [54.64-63.11] | 0.835 |
|  | Posttreatment | 61.20 [55.30-74.57] | 59.46 [53.92-66.98] | 61.65 [60.74-77.10] | 0.676 |
|  | Change | 4.72 [-5.46-12.77] | -4.07 [-5.92-7.15] | 10.35 [2.30-15.74] | 0.421 |
|  | Change Percent（%） | 8.80 [-9.35-23.83] | -5.73 [-11.88-13.67] | 24.94 [3.94-26.27] | 0.151 |
| QTc | Pretherapy | 421.50 [394.85-433.51] | 413.72 [402.27-429.81] | 429.29 [391.24-434.75] | 0.835 |
|  | Posttreatment | 424.78 [406.74-428.69] | 416.91 [403.35-422.48] | 427.90 [427.09-432.31] | 0.296 |
|  | Change | -1.97 [-9.61-26.14] | -7.33 [-10.37-24.53] | -1.39 [-2.54-37.69] | 0.463 |
|  | Change Percent（%） | -0.45 [-2.31-6.54] | -1.71 [-2.51-6.25] | -0.32 [-0.58-9.68] | 0.548 |

WBC: Leukocyte, PLT: Platelet, HGB: Hemoglobin, ALT: Alanine Aminotransferase, AST: Aspartate Aminotransferase, TBIL: Total Bilirubin, BUN: Urea nitrogen, CREA: Creatinine.

Mann-Whitney U-test was used for the comparison of WBC, PLT, HGB, ALT, AST, TBIL, BUN, CREA, and ECG QTc interval between Linezolid and Contezolid group. Change = Posttreatment - Pretherapy. Change Percent（%）= (Posttreatment - Pretherapy)/Pretherapy

**Original data**

Contezolid and Linezolid concentration in CSF and Blood

| group | time points | patients 1 | patients 2 | patients 3 | patients 4 | patients 5 |
| --- | --- | --- | --- | --- | --- | --- |
| Contezolid concentration in CSF | 2h | 1.0643 μg/mL | 1.1566 μg/mL | 1.0806 μg/mL | 1.3165 μg/mL | 0.9295 μg/mL |
|  | 6h | 0.8166 μg/mL | 0.1867 μg/mL | 0.4567 μg/mL | 1.0194 μg/mL | 0.792 μg/mL |
| Contezolid concentration in blood | 2h | 9.241 μg/mL | 25.784 μg/mL | 12.594 μg/mL | 14.694 μg/mL | 8.298 μg/mL |
|  | 6h | 6.056 μg/mL | 3.858 μg/mL | 5.394 μg/mL | 11.21 μg/mL | 5.59 μg/mL |
| Linezolid concentration in CSF | 2h | 1.9545 μg/mL | 2.4154 μg/mL | 4.9636 μg/mL | 3.442 μg/mL | 3.251 μg/mL |
|  | 6h | 1.623 μg/mL | 0.941 μg/mL | 1.752 μg/mL | 1.222 μg/mL | 1.765 μg/mL |
| Linezolid concentration in blood | 2h | 13.1341 μg/mL | 7.8651 μg/mL | 24.666 μg/mL | 22.9138 μg/mL | 21.7999 μg/mL |
|  | 6h | 7.3385 μg/mL | 5.8773 μg/mL | 10.3957 μg/mL | 7.9311 μg/mL | 9.7314 μg/mL |

****Basic data and safty evaluation****

| **group** | **patients** | **sex** | **age** | **BMI** | **CSF** | | **WBC** | | **PLT** | | **HGB** | | **ALT** | | **AST** | | **TBIL** | | **BUN** | | **Crea** | | **QTc** | |
| --- | --- | --- | --- | --- | --- | --- | --- | --- | --- | --- | --- | --- | --- | --- | --- | --- | --- | --- | --- | --- | --- | --- | --- | --- |
|  |  |  |  |  | **WBC** | **N%** | **pre** | **post** | **pre** | **post** | **pre** | **post** | **pre** | **post** | **pre** | **post** | **pre** | **post** | **pre** | **post** | **pre** | **post** | **pre** | **post** |
| Linezolid | 1 | f | 40 | 19.11 | 575 | 5 | 6.67 | 4.24 | 324 | 242 | 135 | 128 | 28.8 | 17.1 | 15.1 | 32.7 | 13 | 12 | 3.10 | 2.88 | 71 | 67 | 445 | 390 |
| Linezolid | 2 | f | 53 | 18.52 | 315 | 6 | 5.84 | 6.55 | 315 | 227 | 147 | 110 | 30.2 | 16.0 | 30.5 | 28.0 | 7 | 16 | 4.04 | 5.65 | 66 | 80 | 430 | 422 |
| Linezolid | 3 | f | 57 | 17.25 | 201 | 10 | 8.46 | 3.05 | 238 | 273 | 100 | 109 | 12.1 | 16.3 | 24.0 | 22.4 | 5 | 8 | 4.88 | 2.58 | 52 | 59 | 402 | 429 |
| Linezolid | 4 | m | 41 | 17.85 | 486 | 6 | 7.96 | 4.08 | 335 | 186 | 134 | 128 | 9.4 | 28.6 | 14.7 | 23.2 | 7 | 6 | 3.77 | 3.83 | 50 | 44 | 392 | 417 |
| Linezolid | 5 | m | 43 | 17.63 | 439 | 9 | 7.52 | 4.68 | 360 | 265 | 149 | 79 | 17.1 | 14.1 | 36.8 | 15.3 | 13 | 11 | 2.34 | 6.43 | 61 | 54 | 414 | 403 |
| Contezolid | 1 | **m** | 29 | 17.5 | 322 | 5 | 11.93 | 8.06 | 229 | 197 | 137 | 111 | 5.6 | 17.7 | 33.1 | 26.1 | 9 | 12.5 | 5.8 | 2.3 | 63.1 | 78.9 | 391.2 | 435.2 |
| Contezolid | 2 | **m** | 63 | 18.4 | 429 | 8 | 8.24 | 6.82 | 330 | 218 | 140 | 108 | 4.0 | 13.7 | 22.7 | 15.3 | 7 | 7.7 | 2.9 | 4.7 | 58.4 | 60.7 | 434.9 | 432.3 |
| Contezolid | 3 | **m** | 58 | 18.2 | 162 | 6 | 5.04 | 2.93 | 451 | 252 | 116 | 138 | 4.0 | 10.5 | 18.4 | 24.7 | 8 | 7 | 4.14 | 2.32 | 69 | 62 | 389 | 427.1 |
| Contezolid | 4 | **f** | 37 | 17.9 | 436 | 11 | 9.22 | 6.89 | 234 | 250 | 124 | 98 | 19.4 | 18.4 | 20.8 | 18.4 | 5 | 13 | 4.07 | 2.42 | 55 | 77 | 435 | 380.0 |
| Contezolid | 5 | **f** | 65 | 16.5 | 608 | 9 | 5.60 | 3.71 | 229 | 196 | 78 | 115 | 4.0 | 22.9 | 30.3 | 11.0 | 10 | 6 | 6.53 | 4.70 | 39 | 50 | 429 | 427.9 |
